# Genome-Wide Analysis of *CSL* Family Genes Involved in Petiole Elongation, Floral Petalization, and Response to Salinity Stress in *Nelumbo nucifera*

**DOI:** 10.3390/ijms252312531

**Published:** 2024-11-22

**Authors:** Jie Yang, Juan Wang, Dongmei Yang, Wennian Xia, Li Wang, Sha Wang, Hanqian Zhao, Longqing Chen, Huizhen Hu

**Affiliations:** Yunnan Province Engineering Research Center for Functional Flower Resources and Industrialization, College of Landscape Architecture and Horticulture Sciences, Southwest Forestry University, Kunming 650224, China; 18212757119@swfu.edu.cn (J.Y.); 24511wj@swfu.edu.cn (J.W.); donna_0507@swfu.edu.cn (D.Y.); sharon@swfu.edu.cn (W.X.); wangli@swfu.edu.cn (L.W.); alizarin_826@swfu.edu.cn (H.Z.)

**Keywords:** cellulose synthase-like family genes, expression pattern, petiole elongation, floral petalization, salinity stress, lotus (*N. nucifera*)

## Abstract

Lotus (*Nelumbo nucifera*), a perennial aquatic plant, endures various environmental stresses. Its diverse ornamental traits make it an ideal model for studying multigene family functional differentiation and abiotic stress responses. The *cellulose synthase-like* (*CSL*) gene family includes multiple subfamilies and holds potentially pivotal roles in plant growth, development, and stress responses. Thus, understanding this family is essential for uncovering the attributes of ancient dicotyledonous lotus species and offering new genetic resources for targeted genetic improvement. Herein, we conducted a genome-wide *NnCSL* gene identification study, integrating tissue-specific expression analysis, RNA-seq, and qRT-PCR validation. We identified candidate *NnCSL* genes linked to petiole elongation, floral petalization, salinity stress responses, and potential co-expressed TFs. 22 *NnCSL* genes were categorized into six subfamilies: NnCSLA, NnCSLB, NnCSLC, NnCSLD, NnCSLE, and NnCSLG. Promoter regions contain numerous cis-acting elements related to growth, development, stress responses, and hormone regulation. Nineteen NnCSL genes showed specific differential expression in LPA (large plant architecture) versus SPA (small plant architecture): petioles, petalized carpels (CP) and normal carpels (C), and petalized stamens (SP) and normal stamens (S). Notably, most *NnCSLC*, *NnCSLA*, and *NnCSLB* subfamily genes play diverse roles in various aspects of lotus growth and development, while *NnCSLE* and *NnCSLG* are specifically involved in carpel petalization and petiole elongation, respectively. Additionally, 11 candidate *NnCSL* genes responsive to salinity stress were identified, generally exhibiting antagonistic effects on growth and developmental processes. These findings provide an important theoretical foundation and novel insights for the functional study of *NnCSL* genes in growth, development, and stress resistance in lotus.

## 1. Introduction

The plant cell wall is a sophisticated and dynamic structure, predominantly classified into two distinct types: the primary cell wall and the secondary cell wall. The primary cell wall is crucial for cell elongation and division, whereas the secondary cell wall provides mechanical support necessary for growth. Both types are integral to plant development and the plant’s response to environmental stressors [1,2,3,4,5]. The cell wall is composed of three main polysaccharides: cellulose, hemicelluloses, and pectic polysaccharides [4]. Composed of linear chains of β-D-glucans interconnected by (1–4) glycosidic bonds, cellulose forms the structural backbone of the cell wall [6]. Hemicelluloses are a diverse group of polysaccharides, including xylans, xyloglucans, mannans, β-(1→3,1→4)-glucans (MLG), and glucomannans [7]. These polysaccharides fortify the cell wall by interacting synergistically with cellulose and, in certain instances, lignin. Studies have demonstrated that these function as the dominating backbone and linkers in the cell wall, with cellulose microfibrils providing tensile strength and hemicelluloses acting as cross-linking agents, creating a network structure that is vital for maintaining the mechanical strength and structural integrity of plant stems [8,9,10,11,12]. Pectin, primarily composed of rhamnogalacturonan I and homogalacturonan (HG), alongside minor constituents such as arabinan, xylogalacturonan, arabinogalactan I, and rhamnogalacturonan II, plays a crucial role in occupying the spaces between hemicelluloses and cellulose, thereby contributing to the stabilization of the cell wall matrix [13,14].

The *cellulose synthase-like* (*CSL*) gene family includes multiple subfamilies—CSLA, CSLB, CSLD, CSLC, CSLE, CSLF, CSLG, CSLH, CSLJ, and CSLM—each belonging to the glycosyltransferase-2 (GT2) superfamily and typically characterized by catalytic domains featuring the D, D, D, and QXXRW motifs. Notably, CSL genes exhibit high-sequence homology with the *cellulose synthase* (*CESA*) genes [3,15,16,17]. Among these subfamilies, CSLA, CSLC, CSLD, and CSLE are uniformly distributed across monocots and dicots, whereas CSLB, CSLG, and CSLM are specific to dicotyledons (eudicots). Conversely, CSLF, CSLH, and CSLJ are restricted to monocots (Poaceae) [16,18,19]. Studies have shown that the CSL family is primarily involved in the synthesis of the backbones of non-cellulosic polysaccharides, including glucuronoarabinoxylan, xyloglucan, galactoglucomannan, and MLG, and the β-galactan side chains of arabinogalactan proteins [20,21,22,23,24,25,26,27]. This pivotal role in polysaccharide synthesis significantly influences plant growth, development, and stress responses, thereby elucidating the functional diversity and evolutionary divergence within the CSL gene family.

The CSLD subfamily, closely related to the CESA family, has been extensively researched. CSLD proteins are pivotal in synthesizing xylan and homogalacturonan, and occasionally cellulose or mannan in tip-growing cells [17,28,29,30,31,32,33]. These genes can be categorized into three functional groups: (i) genes required for pollen germination and tube elongation, such as *AtCSLD1*, *AtCSLD4*, and *PbrCSLD5* [34,35]; (ii) genes predominantly expressed in roots, with mutations resulting in root defects, such as *AtCSLD2*, *AtCSLD3*, *OsCSLD1*, *ZmCSLD5*, *GhCSLD3*, and *LjCSLD1* [31,34,36,37,38,39,40]; (iii) genes affecting leaf morphology and plant architecture, including *AtCSLD5*, *OsCSLD4*, *ZmCSLD1*, and *GhCSLD6* [28,41,42,43,44,45]. Some of these genes also affect cell division [43,46]. Moreover, *AtCSLD5* and *OsCSLD4* also play a vital role in osmotic stress tolerance [47,48]. Thus, the CSLD subfamily represents a crucial set of genes with multifaceted roles in various aspects of plant development and environmental adaptation.

The CSLA and CSLC subfamilies are closely related yet distinct from the CESA family [49]. Early biochemical investigations have demonstrated that the CSLA encodes β-1,4-mannan synthase, essential for mannan synthesis, including glucomannan [50,51]. In *Populus trichocarpa*, *PtCSLA*1 and *PtCSLA3* were identified as mannan synthases, with *PtCSLA1* also functioning as a glucomannan synthase critical for producing (1/4)-β-D-glucomannan, particularly in xylem tissue [16]. In *Arabidopsis*, studies using *csla9* and triple mutants *csla2csla3csla9* confirmed the role of *AtCSLA* glycosyltransferases in glucomannan synthesis [52]. In *Dendrobium officinale*, *DofCSLA14* and *DofCSLA15* were highly expressed in stems, suggesting their critical role in mannan synthesis [24]. Unconventionally, recent investigations have unveiled a striking positive correlation between the expression of *ZjCESA1* and *ZjCSLA1* and the activity of cellulose synthase. Temporary overexpression of either *ZjCesA1* or *ZjCSLA1* in *jujube* fruits led to an elevation in both cellulose synthase activity and cellulose content. This suggests that *ZjCSLA* plays a crucial role in cellulose biosynthesis during fruit development in *Ziziphus jujuba* [26]. The CSLC subfamily, on the other hand, encompasses enzymes that are integral to the formation of the 1,4-β-glucan backbone of xyloglucans and other polysaccharides in *Arabidopsis* [53]. Recent findings highlight that *OsCSLC2* and its homologs are crucial for ethylene-mediated xyloglucan biosynthesis, particularly in the cell walls of root epidermal cells in rice [54]. A deficiency in xyloglucan leads to reduced turgor pressure and altered cell wall properties, negatively impacting early seedling establishment in *Arabidopsis* [55].

Research on monocotyledonous plants, such as rice, barley, sorghum, and *Brachypodium distachyon*, has revealed that the synthesis of MLG is mediated by proteins belonging to the CSLF and CSLH subfamilies [19,27,56,57,58,59]. Importantly, the overexpression of *HvCSLF3* and *HvCSLF10* genes has been found to produce a novel linear glucoxylan consisting of (1,4)-β-linked glucose and xylose residues [59]. The heterologous expression of *HvCSLF3* in wild-type and root-hair-deficient *Arabidopsis* mutants (*csld3* and *csld5*) demonstrated that this gene could compensate for the *csld5* mutant phenotype, indicating that members of the *CSLD* and *CSLF* gene families have similar roles in regulating root growth [60]. This suggests that CSLF is involved in forming not only MLG but also (1,4)-β-glucosidic and (1,4)-β-xylosidic linkages. In contrast, the functions of the CSLB, CSLE, CSLG, and CSLM subfamilies remain poorly understood [22].

In summary, *CSL* family genes are integral to plant growth, development, and stress responses by mediating the synthesis of cellulosic polysaccharides, but current research is still limited. Lotus, a perennial aquatic herbaceous plant, stands out due to its diverse plant architecture, flower colors, and forms, making it an exemplary model for studying the functional differentiation within multigene families [61]. Moreover, as an ancient dicotyledon that retains certain monocot traits, lotus offers valuable insights for evolutionary and taxonomic investigations [62]. This perennial aquatic plant faces various environmental stresses, including salinity, alkalinity, low temperatures, and waterlogging [48,63,64]. Given this, this study aims to elucidate the dicotyledonous and monocotyledonous attributes of lotus through the conserved multigene family of CSL. Concurrently, it seeks to comprehensively elucidate the functions of various *CSL* subfamily genes in lotus, with particular emphasis on those subfamilies that have been less extensively studied, such as CSLB, CSLE, CSLG, and CSLM. Consequently, a comprehensive genome-wide identification of *NnCSL* family genes in lotus was performed, coupled with the investigation of candidate *NnCSL* genes associated with key ornamental traits, including petiole elongation and floral petalization. Additionally, the analysis of their responses to salinity stress along with potentially co-expressed TFs aims to establish a theoretical foundation for further studies. This not only enhances the understanding of lotus growth and stress tolerance but also offers novel insights into the functional investigation of *CSL* family genes, thereby providing new genetic resources for the targeted genetic improvement of lotus.

## 2. Results

### 2.1. Identification of CSL Family Members in N. nucifera

To identify all *CSL* genes in lotus (*N. nucifera*), we employed BLASTP and Hidden Markov Models (HMM). By integrating data from the Conserved Domain Database (CDD) and using batch conservative domain search tools, we identified gene sequences containing the Cellulose_synt (PF03552) and GT2 (PF00535) conserved domains, leading to the discovery of 22 *NnCSL* candidate genes (Table 1). These genes were named mainly based on their closer phylogenetic relationships with *Arabidopsis* and their chromosomal locations, resulting in the following designations: *NnCSLA1*/*2*, *NnCSLB1*/*2*, *NnCSLC1/2/3/4/5*, *NnCSLD1*/2/3/4/5, *NnCSLE1*/2/3/4/5, and *NnCSLG1/2/3.*

The basic information, including genomic length, amino acid residues, molecular weight (Mw), isoelectric point (PI), instability index, grand average of hydropathy (GRAVY), transmembrane domains (TMHs), and subcellular localization, was predicted (Table 1 and Table S1). Specifically, physicochemical analysis revealed that the open reading frame (ORF) lengths of these 22 *NnCSL* family genes ranged from 1425 bp (*NnCSLE3*) to 3456 bp (*NnCSLD3*). Mw varied between 61.230 kDa (*NnCSLA2*) and 129.122 kDa (*NnCSLD3*). The PI ranged from 5.86 (*NnCSLB2*) to 9.11 (*NnCSLA2*), with 27% of the proteins exhibiting a PI below 7, indicating weak acidity. Overall, 68% of NnCSL proteins exhibited relatively low stability. The GRAVY values ranged from −0.02 (*NnCSLE1*) to 0.208 (*NnCSLA1*), with 36% of proteins classified as hydrophilic and the remainder as hydrophobic. The number of TMHs varied from 2 (*NnCSLB2*) to 8 (*NnCSLB1*, *NnCSLD2*, *NnCSLD4, NnCSLE1*, *NnCSLE5*, *NnCSLG2*, and *NnCSLG3*). Subcellular localization analysis confirmed that all identified NnCSL proteins are localized to the plasma membrane.

### 2.2. Phylogenetic Analysis and Classification of the NnCSL Family

**Figure 1:**
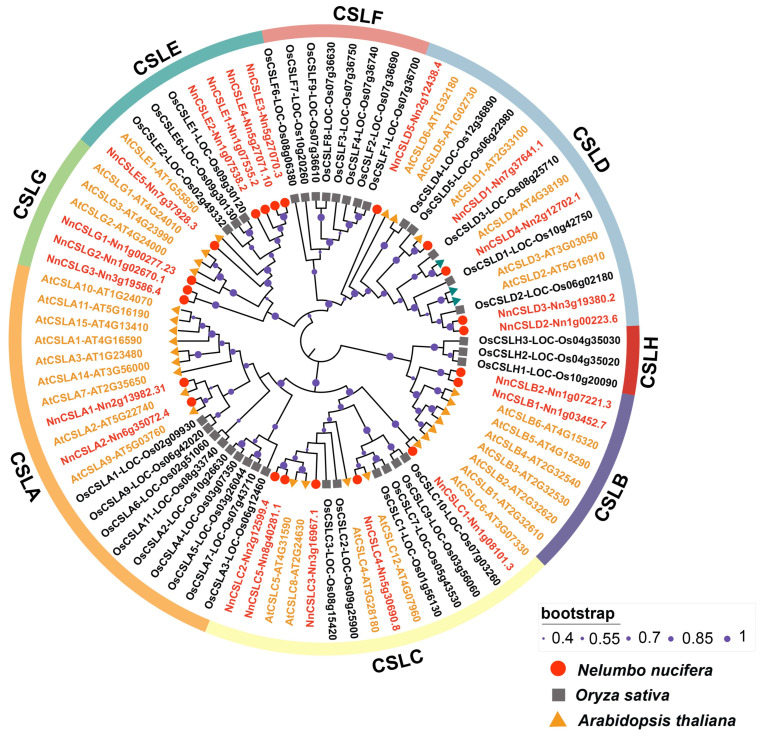
Phylogenetic tree of CSL proteins from *N. nucifera* (Nn), *A. thaliana* (At), and *O. sativa* (Os), using the neighbor-joining method. The eight CSL subfamilies (CSLA, CSLB, CSLC, CSLD, CSLE, CSLF, CSLG, CSLH) are indicated by different circle colors. Red circles denote NnCSL, orange triangles denote AtCSL, and black squares denote OsCSL. Bootstrap values are shown as colored points along the branches.

To elucidate the evolutionary relationships of the *CSL* gene family in *N. nucifera*, we constructed a phylogenetic tree using a total of 86 full-length protein sequences, including 30 *Arabidopsis thaliana CSL* genes (*AtCSL*), 34 *Oryza sativa CSL* genes (*OsCSL*), and 22 *N. nucifera CSL* genes (*NnCSL*) (Figure 1 and Tables S3–S5). The phylogenetic analysis revealed two principal branches of *CSL* genes: one branch comprised the *CSLA*, *CSLC*, *CSLB*, and *CSLH* subfamilies, with *CSLA* and *CSLC* showing higher homology; the other branch included the *CSLD*, *CSLF*, *CSLE*, and *CSLG* subfamilies, with *CSLE* and *CSLG* exhibiting higher homology. In detail, the CSLA subfamily included *AtCSLA1/2/3/4/5/6/7/8/9/10/11/12/13/14/15*, *OsCSLA1/2/3/4/5/6/7/8/9,* and *NnCSLA1/2*; the CSLC subfamily consisted of *AtCSLC4/5/6/7/8/9/10/11/12*, *OsCSLC1/2/3/4/5/6/7/8/9/10*, and *NnCSLC1/2/3/4/5*; the CSLD subfamily was represented by *AtCSLD1/2/3/4/5/6*, *OsCSLD1/2/3/4/5*, and *NnCSLD1/2/3/4/5*; and the CSLE subfamily included *AtCSLE1*, *OsCSLE1/2/3/4/5/6*, and *NnCSLE1/2/3/4/5*. These four subfamilies were common to all three species. The CSLB subfamily comprised *AtCSLB1/2/3/4/5/6/7/8* and *NnCSLB1/2*, while the CSLG subfamily consisted of *AtCSLG1/2/3* and *NnCSLG1/2/3*. These two subfamilies were specific to dicotyledonous plants. Conversely, CSLF and CSLH subfamilies were identified in monocotyledonous plants, including *OsCSLF1/2/3/4/5/6/7/8* and *OsCSLH1/2/3*, respectively [18].

### 2.3. Structure Analysis of CSL Family Members in N. nucifera

**Figure 2:**
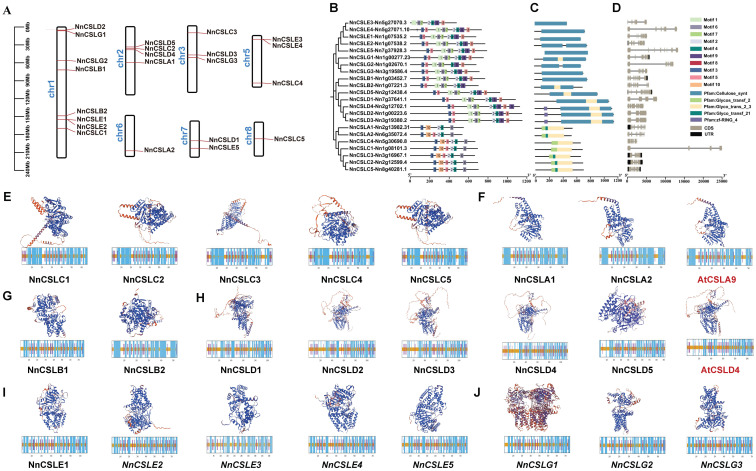
Analysis of *NnCSL* family members: chromosome localization, conserved motifs, gene domains, gene and protein structure. (**A**) Chromosome localization of 22 *NnCSL* genes. The scale provided represents the chromosome size (Mbp). (**B**) Motif composition of *NnCSL* genes, with distinct motifs shown as colored boxes, as indicated in the scheme on the right. (**C**) Conserved domain, represented by different color boxes. (**D**) Exon–intron structure of *NnCSL* genes, with UTRs and CDS indicating untranslated regions and coding sequences, respectively. (**E**–**J**) Secondary and 3D structure predictions for 22 NnCSL proteins, AtCSLA9, and AtCSLD4. The top images display the 3D structures, while the bottom images show the secondary structures. In the secondary structure diagrams, blue, purple, and brown represent alpha helices, extended strands, and random coils, respectively. In the 3D structure visualizations, blue indicates a consistency greater than 70%, yellow represents a consistency between 60% and 70%, and red signifies a consistency of less than 50%.

The genomic annotation data of lotus indicated that 22 *NnCSL* family genes were primarily distributed across seven chromosomes (Chr): Chr1, Chr2, Chr3, Chr5, Chr6, Chr7, and Chr8 (Figure 2A). However, this distribution was uneven, with gene counts on these chromosomes being 8, 4, 3, 3, 1, 2, and 1, respectively. This uneven distribution could be due to non-uniform duplication events occurring across chromosomal segments. The distribution patterns of genes among various CSL subfamilies also showed significant variability. Specifically, the *NnCSLB* subfamily genes were exclusively located on Chr1, while other *CSL* subfamily genes were more dispersed. For instance, *NnCSLA* subfamily genes were found on Chr2 and Chr6; the *NnCSLG* subfamily genes were present on Chr1 and Chr3; the *CSLC* subfamily genes were dispersed across Chr1, Chr2, Chr3, Chr5, and Chr8; the *CSLD* subfamily genes were located on Chr1, Chr2, Chr3, and Chr7; the *CSLE* subfamily genes were distributed on Chr1, Chr5, and Chr7. These observations indicated that the distribution of *CSL* genes in lotus is influenced not only by the overall genomic structure but also by the functional evolution of each subfamily.

To explore the structural features of NnCSL proteins, motif identification was conducted using the MEME tool. It was observed that proteins within the same subfamily generally exhibited similar types and numbers of motifs, with comparable distribution patterns. In total, 10 distinct conserved motifs were identified among the NnCSL proteins, with each protein containing between 5 and 10 motifs (Figure 2B). Specifically, proteins from the NnCSLD, NnCSLB, NnCSLE, and NnCSLG subfamilies—except for NnCSLE3, NnCSLG2, and NnCSLB2—harbored 9 common motifs (motifs 1 through 9). In contrast, proteins from the CSLA and CSLC subfamilies shared only 5 motifs (motifs 2, 3, 4, 6, and 10), with motif 10 being unique to these subfamilies.

A domain analysis of the 22 NnCSLs identified a total of 5 conserved domains (Figure 2C). The genes in the NnCSLA and NnCSLC subfamilies (excluding NnCSLC1 and NnCSLC4) shared the same domains: Glycos_transf_2 (PF00535), Glyco_trans_2_3, and Glyco_transf_21. In contrast, genes from the NnCSLB, NnCSLD, NnCSLE, and NnCSLG subfamilies contained the cellulose synthase domain—Cellulose_synt (PF03552). This suggests that NnCSLs within the same subfamily have similar domain features. Additionally, the zinc finger domain was specifically present only in NnCSLD2, NnCSLD3, and NnCSLD4.

Furthermore, an analysis of the intron-exon structures of *NnCSL* genes revealed the following patterns (Figure 2D). Genes within the *NnCSLA* and *NnCSLB* subfamilies (excluding *NnCSLB2*) consistently had 9 exons. In contrast, genes from the *NnCSLD* subfamily displayed the greatest variability, with exon numbers ranging from 3 to 11. The *NnCSLC* subfamily genes had the fewest exons, ranging from 4 to 6. This analysis underscores the similarity in intron and exon numbers and distributions within the same subfamily, highlighting the conserved nature of NnCSL structures.

The secondary structure of proteins comprises α-helices, β-sheets, β-turns, extended chains, and random coils. Predictions of the secondary structure of 22 NnCSL proteins using the SOPMA tool (Figure 2E–J lower rows and Table S1) revealed that the NnCSL protein sequences primarily consist of α-helices, extended chains, and random coils, with comparable proportions of each. Notably, α-helices and random coils were dominant, while extended chains were less common, and β-sheets were absent.

Additionally, we predicted the tertiary structure of NnCSL family proteins using the Phyre2 threading method (de novo modeling). The results indicated significant variability in their three-dimensional structures (Figure 2E–J upper rows). Among them, proteins from the NnCSLA and NnCSLD subfamilies exhibited relatively high structural similarity within their respective groups. Specifically, NnCSLA1 and NnCSLA2 exhibited homologies of 62.61% and 75.5% with AtCSLA9, respectively. Within the NnCSLD subfamily, NnCSLD3 and NnCSLD4 demonstrated the closest structural similarity to AtCSLD4, with homologies of 67.85% and 75.8%, respectively. This suggests functional differentiation among NnCSL proteins. The validation of tertiary structure conformations using PDBsum Generate (Table S2) revealed that, except for NnCSLC3, all other NnCSL proteins had less than 1% of amino acids in the disallowed regions, indicating stable spatial structures. Moreover, the proportion of amino acids in the favored and allowed regions exceeded 88% for all NnCSL proteins, with most having over 90% in the most favored regions, suggesting reasonable conformations. All NnCSLs exhibited a generation factor value greater than −0.5, indicating normal spatial structures and potentially functional biological roles.

### 2.4. Cis-Acting Elements and Transcription Factor Binding Sites Analysis in the Promoter Region of NnCSL Family Genes

**Figure 3:**
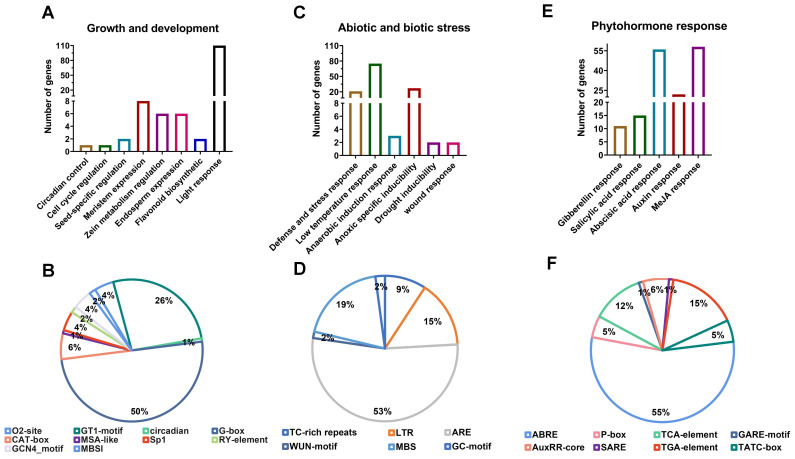
Analysis of cis-regulatory elements in the *NnCSL* promoter regions. (**A**,**C**,**E**) Counts of *NnCSL* genes associated with growth and development, abiotic and biotic stresses, and phytohormone responses. (**B**,**D**,**F**) Pie charts depicting the proportion of different cis-elements in each category: growth and development as shown in (**A**), abiotic and biotic stresses as shown in (**C**), and phytohormone responses as shown in (**E**). Different colors in the pie charts represent various cis-regulatory elements and their proportions in *NnCSL* genes.

Promoters play a pivotal role in determining gene expression at the transcriptional level, with their regulation largely contingent upon cis-acting elements located upstream of the genes. Therefore, we extracted the 2000 bp upstream regions of *NnCSL* genes to identify their putative cis-elements (Figure 3 and Figure S1). We identified three types of cis-acting elements: growth and development-related elements, stress response elements, and hormone response elements. For growth and development-related elements (Figure 3A and Figure S1), we identified eight types of relevant elements, including light response, meristem expression, endosperm expression, and zein metabolism. The key motifs (Figure 3B) included G-box (50%), GT1 (26%), CAT-box (6%), O2-site (4%), Sp1 (4%), and GCN4 (4%). Regarding stress response elements (Figure 3C and Figure S1), we detected seven types of related elements, such as those responding to anaerobic conditions, drought, and low temperature. The key motifs (Figure 3D) included ARE (53%), MBS (19%), LTR (15%), and GC-motif (9%), with most being anaerobic response elements. We also identified five types of hormone response elements (Figure 3E and Figure S1), including MeJA, ABA, and Aux. The main motifs (Figure 3F) were ABRE (55%), TGA-element (15%), and TTCA-element (12%). Transcription factors (TFs) are essential proteins that regulate gene expression by binding to specific DNA sequences (promoter regions) and thus modulate various crucial developmental processes in plants. Accordingly, we further analyzed TF binding sites in the promoter regions of *NnCSL* genes (Figure S2). The analysis revealed a rich array of TF binding sites, with MYB, Dof, and C2H2 being the most abundant. MIKC_MADS, NAC, and WRKY also showed relatively high abundance. Notably, the *NnCSLC4* gene had the highest number of TF binding sites, suggesting that it may have multiple functions in plant growth.

### 2.5. Gene Replication Events and Collinearity Analysis of CSL Family Genes

**Figure 4:**
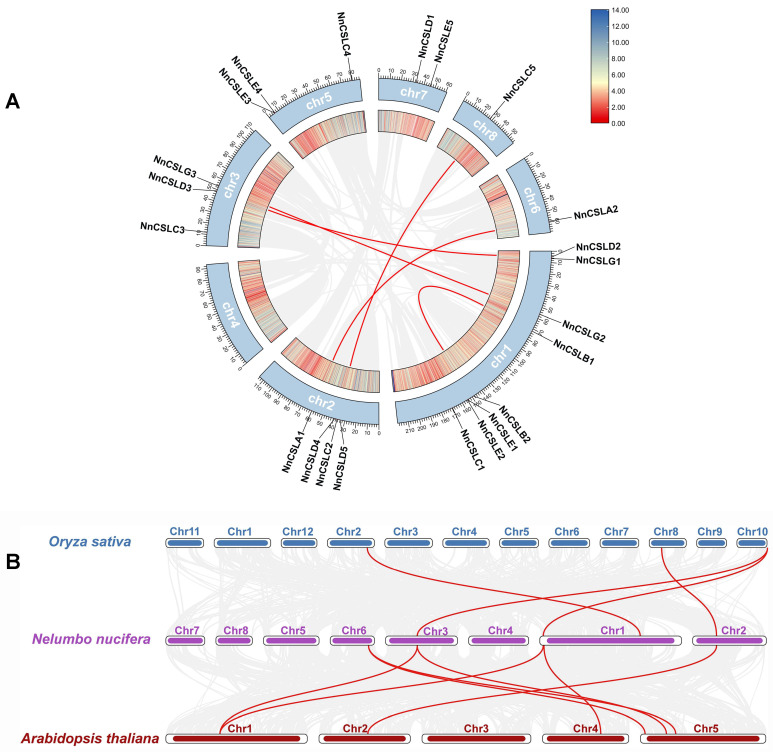
Gene duplication, and synteny of *NnCSL* genes. (**A**) Interchromosomal relationships with red lines indicating segmental gene duplications. (**B**) Synteny analysis of CSL genes among *N. nucifera*, *O. sativa*, and *A. thaliana*, with red lines highlighting syntenic regions.

First, we conducted an intraspecific gene duplication analysis for the 22 *NnCSL* genes. We identified five pairs of segmental duplications on the chromosomes of *N. nucifera*. One pair occurred on Chr1, two pairs were found between Chr1 and Chr3, and the remaining two pairs were located between Chr2 and Chr6, and between Chr2 and Chr8, respectively. Notably, these five pairs of duplicated genes were predominantly within the same subfamilies, such as *NnCSLB1* and *NnCSLB2*, *NnCSLG2* and *NnCSLG3*, and *NnCSLD2* and *NnCSLD3* (Figure 4A). This observation suggests a certain level of conservation within the *NnCSL* subfamilies during the evolutionary process in the *N. nucifera* genome.

Subsequently, we performed comparative syntenic analyses between *N. nucifera* and two representative plant species (*A. thaliana* and *O. sativa*). The analysis identified seven pairs of syntenic homologous genes between *N. nucifera* and *A. thaliana*, and four pairs between *N. nucifera* and *O. sativa*. These *NnCSL* genes were concentrated on Chr1, Chr2, Chr3, and Chr6 in *N. nucifera* (Figure 4B). These findings suggest that the *NnCSL* genes may have similar biological functions to their homologous genes and highlight that *N. nucifera* exhibits more pronounced dicot characteristics, sharing a closer evolutionary relationship with *A. thaliana*.

### 2.6. Tissue Expression Patterns of NnCSL Genes in N. nucifera

**Figure 5:**
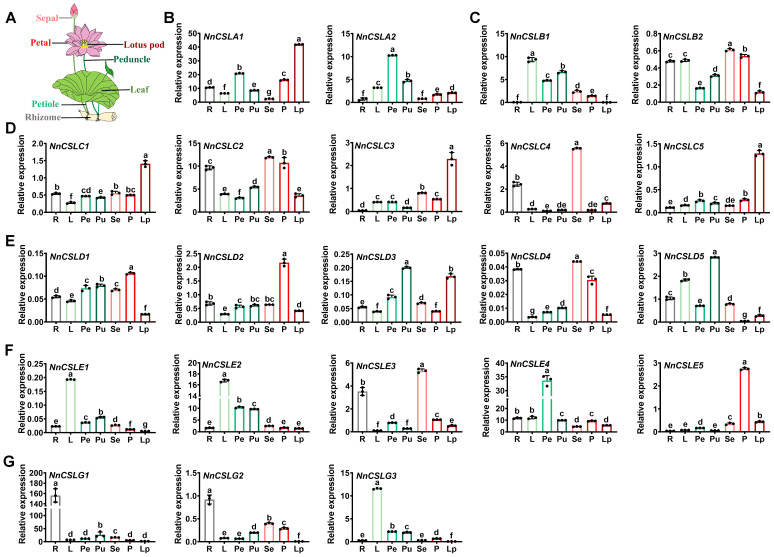
Tissue-specific expression of 22 *NnCSL* genes in *N. nucifera*. R, L, Pe, Pu, Se, P, and Lp represent rhizome, leaves, petioles, peduncles, sepals, petals, and lotus pod, respectively. (**A**) Schematic of the lotus plant showing the tissues analyzed. (**B**–**G**) qRT-PCR analysis of 22 *NnCSL* gene expressions in different tissues. Bars represent means ± SD (n = 3 biological replicates, with individual data points shown). Different letters indicate significant differences (*p* < 0.05) based on one-way ANOVA followed by Tukey’s test. *NnUBC* served as the internal control with its expression value normalized to 100.

Tissue-specific expression profiles can reveal insights into gene function [65,66]. To explore the expression characteristics of *NnCSL* genes across various tissues, including rhizome (R), leaves (L), petioles (Pe), peduncles (Pu), sepals (Se), petals (P), and lotus pods (Lp), qRT-PCR analysis was performed. Rhizomes, leaves, and petioles are categorized as vegetative organs, whereas sepals, petals, and lotus pods are classified as reproductive organs. As illustrated in Figure 5, all 22 genes within the NnCSL family were expressed in both vegetative and reproductive organs. Specifically, genes in the *NnCSLG* subfamily were preferentially expressed in vegetative organs, whereas *NnCSLC* subfamily genes exhibited a dominant expression in reproductive organs, although they were also expressed at relatively high levels in vegetative organs. The expression patterns of *NnCSLA1*, *NnCSLB2*, *NnCSLD1*/*2*/*4*, and *NnCSLE3/5* were similar to those of *NnCSLC* subfamily genes. In contrast, *NnCSLA2*, *NnCSLB1*, *NnCSLD3*/*5*, and *NnCSLE1/2/4* showed the opposite trend of higher expression levels in vegetative organs.

### 2.7. Identification of NnCSL Genes as Candidates for Petiole Elongation in N. nucifera

**Figure 6:**
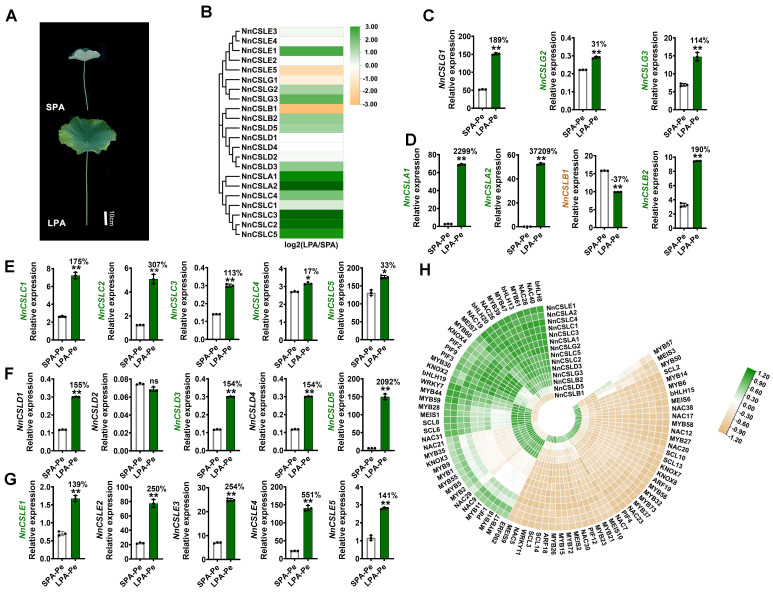
Expression patterns of *NnCSL* genes in petioles of LPA (large plant architecture) and SPA (small plant architecture) lotus varieties. (**A**) Phenotypic comparison of LPA and SPA lotus varieties. (**B**) RNA-seq analysis showing differentially expressed *NnCSL* genes in petioles of LPA and SPA varieties, with expression levels reported as normalized log^2^ FPKM values. (**C**–**G**) qRT-PCR analysis of *NnCSL* gene expression in petioles of LPA and SPA varieties, with *NnUBC* as the internal control (normalized to 100). Data represent means ± SD of three biological replicates. * and ** indicate significant differences between LPA and SPA varieties (*t*-test, *p* < 0.05 or *p* < 0.01, n = 3). Percentage changes relative to SPA are shown; ns denotes not significant. (**H**) Heatmap of enriched correlations between petiole elongation-related candidate *NnCSL* genes and TFs. Green for positive correlation and brown for negative correlation, where darker colors represent stronger correlations.

Tissue expression patterns indicated that *NnCSL* genes were extensively involved in plant growth and development, with distinct roles in vegetative versus reproductive growth. We conducted RNA-seq on petioles from representative large and small lotus varieties (Figure 6A) to further explore the role of *NnCSL* genes in petiole elongation related to plant architecture. Among the 22 *NnCSL* genes, 18 differentially expressed genes (DEGs) were identified in the petioles of LPA (large plant architecture) versus SPA (small plant architecture) lotus varieties (Figure 6B). Notably, most DEGs, such as *NnCSLC1/2/3/4/5*, *NnCSLA1/2*, *NnCSLB2*, *NnCSLD3*/*5*, *NnCSLG2/3*, and *NnCSLE1* were expressed at significantly higher levels in LPA compared to SPA. These results suggest that *NnCSL* genes play a crucial role in positively influencing petiole elongation.

Further validation of *NnCSL* genes was performed using qRT-PCR (Figure 6C–G). The results indicated that, except for *NnCSLB1* and *NnCSLD2*, all other *NnCSL* genes were expressed at significantly higher levels in large lotus varieties compared to small ones. Among these, *NnCSLC1/2/3/4/5*, *NnCSLA1/2*, *NnCSLB2*, *NnCSLD3*, *NnCSLD5*, *NnCSLG2*/*3*, *NnCSLB1*, and *NnCSLE1* were identified as DEGs in RNA-seq. Therefore, we conclude that, aside from *NnCSLB1*, which negatively impacts petiole elongation, the remaining 13 genes are candidates that positively influence petiole elongation in *N. nucifera*.

Co-expression analysis offers detailed insights into the transcriptional interactions between regulatory factors, which is essential for constructing a comprehensive transcriptional regulatory network [67]. This understanding is crucial for elucidating the biological functions and regulatory mechanisms of the *NnCSL* gene family at the protein level. Utilizing DEGs from RNA-seq, we constructed co-expression networks to investigate the relationships among 14 candidate *NnCSL* genes associated with petiole elongation and various TFs through a correlation-clustering analysis (Figure 6H). The analysis revealed that candidate genes positively mediating lotus petiole elongation, such as *NnCSLA1/2*, *NnCSLC1/2/3/4/5*, *NnCSLD3/5*, *NnCSLE1*, and *NnCSLG2/3*, were significantly positively correlated with TFs like NnNAC19/26/40, NnMYB47, NnWRKY7, and NnbHLH8/13/19/20. In contrast, *NnCSLB1*, which negatively affects petiole elongation, had negative correlations with these TFs. Additionally, *NnCSLB1* was significantly positively correlated with NnMYB6/14, NnbHLH15, NnNAC17, and NnKNOX7/8, while *NnCSLA1/2*, *NnCSLC1/2/3/4/5*, *NnCSLD3/5*, *NnCSLE1*, and *NnCSLG2/3* were negatively correlated with these TFs. These TFs, mainly from the MYB, NAC, and bHLH families, are known to be crucial in developing vegetative organs like leaves, lateral branches, and stems. Therefore, we hypothesize that *NnCSL* family genes regulate petiole elongation and plant architecture through a complex network involving these TFs.

### 2.8. Identification of NnCSL Genes as Candidates for Floral Petalization in N. nucifera

**Figure 7:**
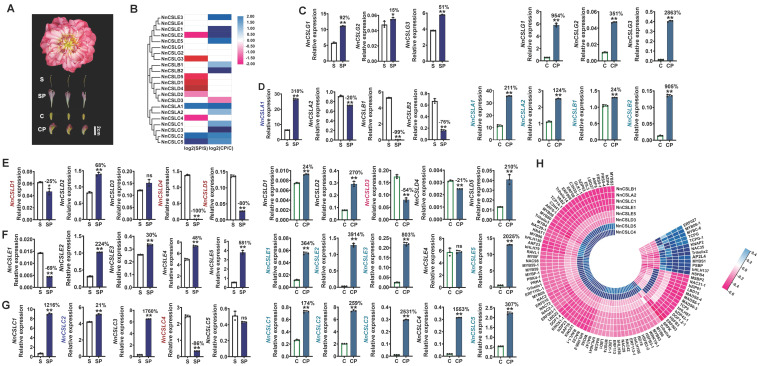
Expression patterns of *NnCSL* genes in floral petalization-related tissues. (**A**) Phenotypic observation of floral structures in duplicate-petalled *N. nucifera*. S, SP, C, and CP denote stamens, stamen petals, carpels, carpel petals, respectively. (**B**) RNA-seq analysis showing differentially expressed *NnCSL* genes related to floral petalization, with expression levels reported as normalized log^2^ FPKM values. (**C**–**G**) qRT-PCR analysis of *NnCSL* gene expression in floral tissues, with *NnUBC* as the internal control (normalized to 100). Data represent means ± SD of three biological replicates. * and ** indicate significant differences between SP and S, or CP and C tissues (*t*-test, *p* < 0.05 or *p* < 0.01, n = 3). Percentage changes relative to S or C are shown; ns denotes not significant. (**H**) Heatmap of enriched correlations between floral petalization-related candidate *NnCSL* genes and TFs. Blue for positive correlation and pink for negative correlation, where darker colors represent stronger correlations.

Previous studies have demonstrated that *CSL* family genes play a role in reproductive processes, including pollen germination and pollen tube growth. Additionally, our observations revealed that numerous *NnCSL* genes are specifically expressed in reproductive organs such as sepals, petals, and lotus seed pods (Figure 5B–G). To further elucidate the role of *NnCSL* family genes in the petalization of stamens and carpels in lotus, we conducted RNA-seq analysis on normal stamens (S), petalized stamens (SP), normal carpels (C), and petalized carpels (CP) of the lotus variety ‘Shen Nvzi’ (Figure 7A). The analysis identified 19 *NnCSL* genes with significant differential expression, excluding *NnCSLE4* and *NnCSLG1/2*. Among these, 10 *NnCSL* genes were differentially expressed between SP and S: *NnCSLC4*, *NnCSLD1*/*2*/*4/5*, and *NnCSLE2* were significantly downregulated in SP, whereas *NnCSLA1*, *NnCSLC2*, and *NnCSLC5* were significantly upregulated in SP compared to S. In the comparison between CP and C, 13 differentially expressed *NnCSL* genes were identified: *NnCSLA1/2*, *NnCSLB1/2*, *NnCSLC1/2/3/5*, and *NnCSLE1/2/3/5* showed significantly higher expression levels in CP, while *NnCSLD3* was downregulated (Figure 7B). Notably, *NnCSLA1* and *NnCSLE2* were significantly upregulated in both SP and CP, with *NnCSLE2* exhibiting higher expression levels in CP but lower expression levels in SP. These findings suggest that *NnCSL* family genes play a crucial role in the petalization of carpels and in maintaining normal stamen development.

qRT-PCR validation further confirmed that, with the exception of *NnCSLC5* and *NnCSLD1/3*, 12 *NnCSL* genes were highly expressed in SP. These included *NnCSLA1*, *NnCSLC1/2/3*, *NnCSLD2*, *NnCSLE2/3/4/5*, and *NnCSLG1/2/3*, with expression levels ranging from 15% (*NnCSLG2*) to approximately 1760% (*NnCSLC3*) higher than in S. Among them, only *NnCSLA1* and *NnCSLC2* were identified as DEGs in RNA-seq. In S, 8 genes—*NnCSLA2*, *NnCSLB1/2*, *NnCSLC4*, *NnCSLD1/4/5*, and *NnCSLE1*—were highly expressed in S, but at levels 25% (*NnCSLD1*) to 100% (*NnCSLD4*) lower than in SP. Notably, *NnCSLC4* and *NnCSLD1/4/5* were also identified as DEGs in RNA-seq. Combining RNA-seq and qRT-PCR results, we identified two candidate genes (*NnCSLA1* and *NnCSLC2*) that may positively mediate stamen petalization, and four candidate genes (*NnCSLC4* and *NnCSLD1/4/5*) that may negatively influence this process in *N. nucifera*. Additionally, except for *NnCSLD3/4* and *NnCSLE4*, all other genes exhibited significantly higher expression levels in CP. These genes—*NnCSLA1/*2, *NnCSLB1/2*, *NnCSLC1/2/3/4/5*, *NnCSLD1/2/5*, and *NnCSLE1/2/3/5*—exhibited expression levels in CP ranging from 24% (*NnCSLB1* and *NnCSLD1*) to 3914% (*NnCSLE2*) higher than in C. The expression patterns of *NnCSLA1*, *NnCSLB2*, *NnCSLC1/2/3/5*, and *NnCSLE2/3/5* were consistent with the RNA-seq data. *NnCSLD3/4* were highly expressed in C, with expression levels more than 21% higher than in CP, and the expression pattern of *NnCSLD3* matched RNA-seq data (Figure 7C–G). Based on these findings, *NnCSLA1*, *NnCSLB2*, *NnCSLC1/2/3/5*, and *NnCSLE2/3/5* were proposed as candidate genes positively involved in carpel petalization, while *NnCSLD3* is suggested as a candidate gene negatively involved in this process.

The analysis of co-expression networks involving 16 candidate *NnCSL* genes (*NnCSLA1*, *NnCSLC2/4*, *NnCSLD1/4/5*, *NnCSLA1*, *NnCSLB2*, *NnCSLC1/2/3/5*, *NnCSLE2/3/5*, and *NnCSLD3*) in relation to floral petalization and TFs revealed significant relationships among these genes (Figure 7H). Specifically, candidate genes involved in petalization, including *NnCSLA2*, *NnCSLB1*, *NnCSLC1*, and *NnCSLE1/5*, exhibited strong positive correlations with TFs such as NnbHLH137, NnERF027, NnNAC35, and NnAP2L4. Conversely, genes predominantly expressed in stamens, namely *NnCSLC4* and *NnCSLD5*, displayed negative correlations with these TFs but were positively correlated with NnARF6-1, NnERF08, NnKNOX1, and NnNAC98. In carpels, the gene *NnCSLD3*, which is highly expressed, showed significant positive correlations with NnbZIP43-1, NnbHLH87, NnMADS6-1, and NnMADS6-3, while demonstrating predominantly negative correlations with other TFs. Other candidate genes did not show significant correlations with the selected TFs.

### 2.9. NnCSL Genes Responding to Salinity Stress Antagonize Growth and Development in N. nucifera

**Figure 8:**
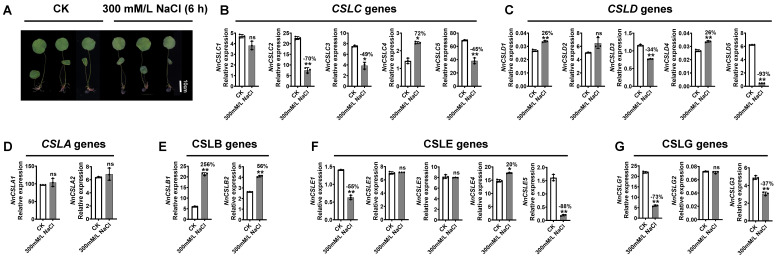
Expression patterns of the *NnCSL* family genes under 300 mM/L NaCl treatment for 6 hours (h). (**A**) Morphological comparison of lotus seedlings treated with 300 mM/L NaCl for 6 h. (**B**–**G**) qRT-PCR analysis of *NnCSL* gene expression in NaCl-treated seedlings versus control (CK), with *NnUBC* as the internal control (normalized to 100). Data represent means ± SD of three biological replicates. * and ** indicate significant differences between NaCl treatment and CK (*t*-test, *p* < 0.05 or *p* < 0.01, n = 3). Percentage changes relative to CK are shown; ns denotes not significant.

Salinity is a prominent abiotic stressor that induces osmotic stress and nutrient imbalances through excessive accumulation of Na^+^, K^+^, and Cl^−^ ions. This can lead to toxic effects during various stages of plant growth, including germination, seedling development, vegetative growth, flowering, and fruit set [68]. To examine whether *NnCSL* family genes exhibit similar responses, lotus seedlings were exposed to 300 mmol/L NaCl for 6 h. The results revealed that many *NnCSL* candidate genes, which are expected to positively impact petiole elongation and carpel petalization, actually responded negatively to salinity stress. This includes genes such as *NnCSLC2/3/5*, *NnCSLG3*, *NnCSLD3/5*, and *NnCSLE1/5* (Figure 8). Specifically, within the *NnCSLC* subfamily, all genes except *NnCSLC4* exhibited a significant decrease in expression after NaCl treatment, with *NnCSLC2* decreasing by over 70% compared to the control. Similarly, the expression levels of *NnCSLD5* and *NnCSLE5* dropped by up to 93%. In contrast, genes in the *NnCSLB* subfamily exhibited a significant positive response to salinity stress. Notably, *NnCSLB1*, which is expressed relatively lower in lotus petioles of LPA, was significantly upregulated under salinity stress. Additionally, *NnCSLD4*, expressed at lower levels in petalized floral organs, also showed significant upregulation. These findings suggest that *NnCSL* genes mediate an antagonistic relationship between salinity stress and plant growth and development.

## 3. Discussion

As one of the multi-gene families in plants, the *CSL* gene family has been extensively characterized in various terrestrial species, including both dicotyledonous and monocotyledonous taxa, such as *A. thaliana*, cotton, *P. trichocarpa*, *D. officinale*, *Z. jujuba*, rice, barley, *Avena sativa*, and sorghum [16,19,24,26,27,53,54,57,58]. However, its presence and roles in the aquatic and ancient species, such as the lotus plant, remain under-examined. Here, we report the genome-wide identification, classification, and expression analysis of *CSL* family genes in lotus. Our findings revealed that 19 *NnCSL* genes exhibit specific differential expression patterns in LPA and SPA petioles, CP and C, and SP and S. Notably, 11 of these genes demonstrated antagonistic responses to salinity stress. Specifically, *NnCSLC2*, *NnCSLA1*, and *NnCSLD3/5* play a significant and broad role in lotus responses to salinity stress, as well as in various growth and developmental processes.

### 3.1. Characteristics of the Lotus CSL Gene Family Indicative of Stronger Dicotyledonous Attributes

In this study, we identified 22 *NnCSL* genes in the lotus, a number that is fewer than those reported in the dicotyledon model *Arabidopsis* (30) and monocot model rice (34), given the relative genome sizes and phylogenetic positions in the species tree. Protein sequence analyses revealed that NnCSLs encompass the PF03552 and PF00535 conserved domains, consistent with the characteristics of the CSL family identified in various species, including *A. thaliana* [22], rice [18], *Z. jujuba* [26], *D. officinal* [24], pineapple [20], and strawberry [25]. Previous studies have indicated that CSLA, CSLC, CSLD, and CSLE are ubiquitous in both monocots and dicots, whereas CSLB, CSLG, and CSLM are dicot-specific, with CSLF, CSLH, and CSLJ being exclusive to monocots [16,18,19]. Here, we identified 22 *NnCSL* family genes, classified into the six subfamilies CSLA, CSLB, CSLC, CSLD, CSLE, and CSLG (Figure 1), consistent with the dicotyledon-like classification observed in *Arabidopsis* [15]. In contrast to rice [18], NnCSLs lack the subfamilies CSLF, CSLH, and CSLJ that are characteristic of monocots, suggesting a more pronounced dicotyledonous attribute in the ancient dicotyledonous lotus. Segmental gene duplication is the dominant factor for generating and maintaining gene families and is also considered the main source of gene structural changes and innovation [69]. Furthermore, comparative syntenic analyses among *N. nucifera*, *A. thaliana*, and *O. sativa* revealed seven syntenic gene pairs between *N. nucifera* and *A. thaliana*, and four pairs between *N. nucifera* and *O. sativa* (Figure 4B). This further indicates that NnCSLs exhibit stronger dicot characteristics, sharing closer evolutionary relationships with AtCSLs. Broadly, homologous genes from different species typically share similar functions [70]. Therefore, the functional study of a homologous gene can provide insights into the function of an unknown gene. Further research is essential to ascertain whether the functions of *NnCSL* genes are analogous to those of their *AtCSL* homologs.

### 3.2. Roles of NnCSLs in Plant Growth and Development

Studies have indicated that *CSL* family genes in dicotyledonous plants primarily contribute to the biosynthesis of various cell wall polysaccharides, including xyloglucan, homogalacturonan, mannan, and cellulose, thereby influencing the composition and structure of the cell wall. These genes are crucial for plant growth, development, and response to biotic and abiotic stresses [20,21,22,23,24,25,26,27,31,32]. However, the functional characterization of *CSL* genes in lotus remains underexplored. Tissue-specific expression profiles can elucidate gene function [65,66]. Previous research has demonstrated that genes like *AtCSLD2* and *AtCSLD3* in *Arabidopsis* [29], *OsCSLD1* in rice [38], *ZmCSLD1* in maize [39], and *LjCSLD1* in *Lotus japonicus* [40] exhibit preferential expression in plant roots, crucial for root-hair development and nutrient absorption. Moreover, *AtCSLD1* and *AtCSLD4* in *Arabidopsis* are highly expressed in mature pollen, and their mutations impair cellulose deposition in pollen tube walls, affecting fertilization [71]. In tobacco, *NaCSLD1* is primarily expressed during pollen maturation and tube growth, regulating flower development [1]. To investigate the role of *NnCSL* genes in plant growth and development, we performed tissue-specific expression analyses, revealing that 22 *NnCSL* genes were expressed in both vegetative and reproductive organs. Notably, genes in the *NnCSLG* subfamily showed preferential expression in vegetative organs, while most *NnCSLC*, *NnCSLA*, and *NnCSLB* subfamily genes were expressed at high levels in both reproductive and vegetative tissues. The *NnCSLD* and *NnCSLE* subfamily genes exhibited distinct expression patterns, with *NnCSLD1/2/4* and *NnCSLE3/5* predominantly expressed in reproductive organs, and *NnCSLD3/5* and *NnCSLE1/2/4* showing higher expression in vegetative organs (Figure 5).

In this study, we analyzed petioles from large and small lotus varieties as representative vegetative organs and included normal and petalized stamens and carpels as reproductive organs. RNA-seq and qRT-PCR analyses revealed that *NnCSL* gene expression patterns were consistent with their tissue-specific profiles (Figure 6 and Figure 7). Specifically, *NnCSLG* (*NnCSLG2/G3*) subfamily genes were predominantly expressed in larger lotus petioles, while *NnCSLE* (*NnCSLE1/E2/E3/E5*) subfamily genes were notably expressed in petalized carpels. Conversely, genes from the *NnCSLC* (*NnCSLC1/C2/C3/C5*), *NnCSLA* (*NnCSLA1/A2*), and *NnCSLB* (*NnCSLB2*) subfamilies exhibited high expression in both larger lotus petioles and petalized carpels. Notably, *NnCSLA1* and *NnCSLC2* were also significantly upregulated in petalized stamens. Additionally, consistent with their expression patterns in vegetative organs (Figure 5), *NnCSLD3* and *NnCSLD5* showed high expression in larger lotus petioles but were primarily expressed in normal stamens and carpels.

Extensive research has been conducted on the CSLA, CSLC, and CSLD subfamilies in dicotyledonous plants, while the functions of the CSLB, CSLE, and CSLG subfamilies remain relatively underexplored [22]. Notably, *CSLA* and *CSLC* genes share a high degree of homology and cluster with CSLB within the same major branch, whereas CSLD, which is closely related to CESA, forms a separate branch with greater sequence similarity to the CSLE and CSLG subfamilies (Figure 1). Our findings suggest that the *NnCSLA*, *NnCSLC*, and *NnCSLB* genes, which group within the same major branch, likely share similar functions related to cell wall polysaccharide synthesis, thereby playing a critical role in the entire plant growth and development period, encompassing both vegetative and reproductive growth. Existing studies indicate that *CSL* genes not only participate in cell wall construction during vegetative growth but also play a crucial role in the reproductive developmental stages of plants. It has been reported that the overexpression of *AtCSLA2*, *AtCSLA7*, and *AtCSLA9* leads to an increase in glucomannan content in stems, which significantly impacts embryonic progression [52]. Research has also shown that *CSLD* genes are involved in the polarized growth of structures such as root hairs and pollen tubes, including *OsCSLD1*, *AtCSLD3*, and *AtCSLD4* [38,71]. Additionally, evidence suggests that the *PbrMADS52–PbrCSLD5* signaling pathway enhances cellulose content in the pear pollen tube cell wall, inhibiting pollen tube growth [35]. Collectively, our findings offer valuable insight into the potential specific roles of *NnCSL* genes in plant growth and development, particularly highlighting the largely unexplored functions of the CSLB, CSLE, and CSLG subfamilies. These subfamilies warrant further investigation to elucidate their specific mechanisms, contributing to a deeper understanding of the diverse roles these genes play in plant biology.

### 3.3. Roles of NnCSLs in Response to Salinity Stress Antagonize Growth and Development

Research has demonstrated that *CSL* family genes not only mediate plant growth and development but also respond to various stressors. In *A. sativa*, the expression of most *AsCSL* family genes were repressed under abiotic stress conditions [27]. Within the *Orchidaceae* family, the CSLA subfamily may play a crucial role in drought stress responses across different life forms, while the CSLD subfamily appears to be essential for epiphytic and saprophytic orchids to adapt to freezing stress [24]. In banana, the genes *MaCSLA4/12*, *MaCSLD4*, and *MaCSLE2* are promising candidates associated with chilling tolerance [23]. In *Fragaria vesca*, the expression levels of numerous *FveCSL* genes were altered following treatment with nordihydroguaiaretic acid [25]. Moreover, CSLD proteins are important for how plants respond to environmental stresses. In *Arabidopsis*, the gene SOS6 encodes a protein called AtCSLD5, and its mutant allele *sos6-1* is crucial for osmotic stress tolerance. Plants with the *sos6-1* mutation are more sensitive to salt stress and osmotic pressure caused by mannitol or polyethylene glycol, as well as to drought conditions [47]. Similarly, knocking out the *OsCSLD4* gene decreases salt and osmotic stress tolerance in rice, while overexpressing *OsCSLD4* improves salt tolerance, thus highlighting its beneficial role in surviving salt stress [48].

Lotus, as a perennial aquatic herbaceous plant, confronts various environmental stresses, including salinity stress [48,63,64]. Consequently, the responsiveness of *NnCSL* family genes to salinity stress and their specific response patterns warrant in-depth investigation. In this study, through the prediction of cis-acting elements in the promoter regions of *NnCSL* genes in lotus, we identified 22 genes containing numerous abiotic stress response elements, suggesting that *NnCSL* genes play a pivotal role in stress responses. Further analysis of the expression changes of *NnCSL* genes under salinity stress revealed that eight genes—*NnCSLC2/3/5*, *NnCSLG3*, *NnCSLD3/5*, and *NnCSLE1/5*—expected to positively impact petiole elongation and carpel petalization, exhibited a negative response to salinity stress. Among them, *NnCSLC2*, *NnCSLD5*, and *NnCSLE5* showed the most pronounced effects (Figure 8). Studies have shown that plants encounter abiotic stresses throughout their development, prompting the production of secondary metabolites to enhance resistance. However, this process demands considerable carbon and nutrients, diverting resources from growth and thereby decelerating development. Consequently, growth and resistance often exhibit antagonistic relationships [72]. This phenomenon could plausibly explain the antagonistic effects observed in our study, where *NnCSLs* response to salinity stress antagonizes growth and development. Nevertheless, the discrepancy between the antagonistic mechanism observed in *NnCSL* genes and the synergistic growth and resistance mediated by *AtCSLD5* and *OsCSLD4* [47,48] highlights species–specific diversity and complexity in stress response strategies and regulatory mechanisms. The underlying mechanisms merit further exploration. This complexity underscores the need for further exploration of the underlying mechanisms, which could provide valuable insights into the adaptive strategies employed by different species under environmental stress conditions.

### 3.4. Potential Complex Regulatory Networks of NnCSLs Involved in Plant Growth and Abiotic Stress Responses

Upstream TFs play a pivotal role in regulating gene expression levels, with the promoter region being essential for this process [73]. Therefore, analyzing cis-acting elements within the promoters of *NnCSL* genes provides an effective approach to predict their regulatory networks. To gain preliminary insights into the regulatory mechanisms of *NnCSL* candidate genes involved in lotus growth and abiotic stress responses, we initially examined TF binding sites within the promoter regions of *NnCSL* genes. The analysis uncovered a diverse array of TF binding sites, including MYB, NAC, and WRKY. Notably, the *NnCSLC4* gene, which harbored the highest number of TF binding sites, showed specific high expression in larger lotus petioles (Figure 6E and Figure S2).

Furthermore, utilizing DEGs from RNA-seq analysis, we constructed co-expression networks based on identified *NnCSL* candidate genes associated with petiole elongation (e.g., *NnCSLA1/2*, *NnCSLC1/2/3/4/5*, *NnCSLD3/5*, *NnCSLE1*, and *NnCSLG2/3*) and those linked to floral petalization (e.g., *NnCSLA1*, *NnCSLC2* and *NnCSLC4*, *NnCSLD1/4/5*; *NnCSLA1*, *NnCSLB2*, *NnCSLC1/2/3/5*, *NnCSLE2/3/5*, and *NnCSLD3*). Notably, TFs such as *NnPIF2/3/9*, *NnKNOX2/4*, *NnMYB61/47/39/60/30/44/59/28*, *NnNAC40/28/26/19*, *bHLH8/13/20/19*, and *NnWRKY7* exhibited significant positive correlations with *NnCSLC2/1/3/5*, *NnCLG2/3*, and *NnCSLD3*, all of which were highly expressed in larger lotus petioles (Figure 6H). Conversely, most TFs exhibited negative correlations with *NnCSL* genes involved in petalization, with the exception of *NnERF027*, *NnMYB06-1/C-6/S1*, *NnAP2L4*, and *NnNAC35*, which positively correlated with *NnCSLE1/5* and *NnCLA2*—genes that were predominantly expressed in petalized carpels (CP) or petalized stamens (SP). These results suggest that NnCSL plays a critical role in modulating both vegetative and reproductive growth through distinct regulatory networks mediated by specific TFs.

Numerous studies have elucidated the pivotal roles of various TFs, including NAC [74,75,76], MYB [77,78], KNOX [74,79], bHLH [80], PIF [81], WRKY [82], and ERF [83], in modulating plant growth, development, and responses to salinity stress. For instance, OsMYB110 has been identified as a direct target of *PHOSPHATE STARVATION RESPONSE 2* (OsPHR2) and plays a crucial role in regulating OsPHR2-mediated suppression of rice height. The inactivation of MYB110 resulted in increased culm diameter and enhanced bending resistance, thereby improving lodging resistance despite the increase in plant height [78]. Furthermore, members of subgroup 19 (SG19) of the R2R3-MYB family have been demonstrated to play essential roles in the later stages of floral organ development and maturation. Extensive research, primarily conducted in *Solanaceae* and *Arabidopsis*, has documented the involvement of these TFs in flower opening [84,85], senescence [84], stamen development [86,87], ovule fertility [85], as well as pistil length and maturation [85,88]. In another context, the *IbMYB308* gene from sweet potato (*Ipomoea batatas*) has been shown to enhance salt stress tolerance in transgenic tobacco [77]. Similarly, *NAC1/NAC2* transcription factors from cowpea have been found to improve growth and tolerance to drought and heat in transgenic cowpea by activating photosynthetic and antioxidant mechanisms [76]. Collectively, these findings lead us to hypothesize that complex regulatory networks involving NnCSLs may exist, influencing plant growth and responses to abiotic stresses. This study, therefore, provides valuable insights that can further elucidate the biological functions and regulatory mechanisms of the *NnCSL* gene family, contributing to the broader understanding of plant growth and stress adaptation.

## 4. Materials and Methods

### 4.1. Plant Materials and Sample Collection

The lotus materials utilized in this study were sourced from the National Lotus Germplasm Repository at Southwest Forestry University, Kunming, China. Tissue samples, encompassing rhizomes, leaves, petioles, peduncles, sepals, petals, lotus pods, stamens, stamen petals, carpels, and carpel petals, were collected during the flowering season in the summer of 2024. Upon collection, samples were immediately flash frozen in liquid nitrogen and stored at −80 °C until further use.

### 4.2. Salinity Stress Treatment

For the salinity stress experiment, seedlings of the lotus variety ‘TKL 36’ were selected as the experimental material. Mature, plump seeds of uniform size were chosen, and the outer shell at the tail end was carefully trimmed. The seeds were then soaked in clean water, with the water changed 1–2 times daily. The soaking process was conducted under full light at 25–30 °C for approximately 15 days, until the first floating leaf was fully expanded. Healthy, uniformly growing seedlings were subsequently transferred to a 300 mM/L NaCl solution, with the water level maintained at about 5 cm, ensuring that the floating leaves remained fully above the water surface. After 6 h of treatment, petioles (3–5 cm below the base of the first floating leaf) were collected. Untreated lotus seedlings served as the control.

### 4.3. Identification and Property Analysis of NnCSLs Family Genes

(1) Data Acquisition: Download the Nelumbo genome, GFF, and protein files from the Nelumbo genome database (http://nelumbo.biocloud.net, accessed on 15 August 2024). Obtain the protein sequences of AtCSLs (Table S3) from the TAIR database (https://www.arabidopsis.org/, accessed on 15 August 2024). Acquire the protein sequences of OsCSLs (Table S4) from the Rice Genome Annotation Database (https://rice.plantbiology.msu.edu/index.shtml, accessed on 15 August 2024). (2) Initial screening with bLAST: Utilize AtCSLs and OsCSLs as seed sequences to perform a bidirectional BLAST search in TBtools, with a threshold set to 10^−5^, for the initial identification of NnCSL proteins. (3) HMM-based validation: Download Hidden Markov Model (HMM) configuration files for Cellulose_synt (PF03552) and Glycos_transf_2 (PF00535) from the Pfam database (http://pfam.xfam.org/, accessed on 18 August 2024). Validate the preliminarily identified NnCSLs protein candidates using these HMM models. Manually curate the sequences, removing those with incomplete domains. The remaining validated sequences represent the NnCSLs family members (Table S5). (4) Protein property analysis: Analyze the amino acid composition, isoelectric point, and molecular weight of the NnCSL proteins using the online tool Protparam (https://web.expasy.org/protparam/, accessed on 21 August 2024). (5) Structure and localization prediction: Predict the transmembrane structures of the NnCSL proteins using TMHMM-2.0 (http://www.cbs.dtu.dk/services/TMHMM/, accessed on 21 August 2024). Determine the subcellular localization of these proteins using WoLF PSORT (https://wolfpsort.hgc.jp/, accessed on 21 August 2024) and CELLO (http://cello.life.nctu.edu.tw/, accessed on 23 August 2024).

### 4.4. Phylogenetic Analysis

As previously described, CSL protein sequences for *A. thaliana* and *O. sativa* were sourced from the TAIR and TIGR databases, respectively, and subsequently combined with the CSL protein amino acid sequences from *N. nucifera*. These compiled sequences were imported into MEGA software (version 7.0) [89] for alignment. Alignment of all CSL proteins from the three species was performed using the MUSCLE algorithm. Phylogenetic analysis was then conducted using the neighbor-joining method, employing the optimal model with 1000 bootstrap replicates for reliability assessment. The resulting phylogenetic tree was visualized and edited using iTOL (https://itol.embl.de/, accessed on 18 November 2024). This streamlined method ensures clarity and consistency, enhancing the reproducibility of the phylogenetic analysis of CSL proteins across the three species.

### 4.5. Chromosome Localization, Gene Structure, Motif Distribution, and Conserved Domains of NnCSL Family Genes

The Gene Location Visualization function in TBtools (V2.056) was utilized to map the *NnCSL* family genes onto their respective chromosomes for visual representation [90] The intron–exon structures of 22 *NnCSL* genes were analyzed using TBtools, leveraging genomic GFF data. Next, conserved motifs within the CSL proteins were examined using the MEME Suite (https://meme-suite.org/meme/, accessed on 5 August 2024), allowing for the identification of up to 10 distinct motifs. Finally, the domain visualization of NnCSL proteins was conducted using the Batch SMART tool within TBtools.

### 4.6. Secondary and 3D Structure Analysis of NnCSL Family Genes

The secondary structure of the NnCSL protein was predicted using SOPMA (https://npsa-prabi.ibcp.fr/cgi-bin/npsa_automat.pl?page=/NPSA/npsa_server.html, accessed on 16 August 2024), which analyzes structural elements such as alpha helices, beta turns, random coils, and extended strands. To analyze the 3D structure of the NnCSL protein, the online tool SWISS-MODEL was employed (https://www.swissmodel.expasy.org/, accessed on 26 August 2024). Additionally, the stability of the 3D structure was assessed using PDBsum (https://www.ebi.ac.uk/msd-srv/prot_int/pistart.html, accessed on 26 August 2024).

### 4.7. Cis-Acting Elements Prediction and Transcription Factor Binding Sites Analysis of NnCSLs Promoter

The Fasta Extract function in TBtools was utilized to obtain a 2000 bp upstream nucleotide sequence fragment (promoter sequence) from the lotus genome file for the NnCSL genes. This fragment was then submitted to the PlantCARE website (http://bioinformatics.psb.ugent.be/webtools/plantcare/html/, accessed on 18 November 2024) and PLACE database (https://www.dna.affrc.go.jp/PLACE/?action=newplace, accessed on 20 November 2024) for the prediction and annotation of cis-acting elements in the *NnCSL* gene promoters, facilitating functional classification and statistical analysis. Ultimately, we utilized the binding site information predicted by the PlantCARE website to generate the graphical representations shown in Figures S1 and S2. Data visualization was conducted using TBtools and GraphPad Prism 5.0. Additionally, the 2000 bp upstream nucleotide sequence fragments of the *NnCSL* genes were submitted to the Plant Transcriptional Regulatory Map (https://plantregmap.gao-lab.org/index-chinese.php, accessed on 17 August 2024) for further prediction and analysis. The prediction results underwent classification and statistical analysis, with data visualization accomplished using the Graphics and Heat Map functions in TBtools.

### 4.8. Gene Duplication and Synteny Analyses

In TBtools, the One Step MCScanX tool [91] was utilized for synteny analysis within individual species and interspecies synteny analysis among *N. nucifera*, *O. sativa*, and *A. thaliana*. Subsequently, the “Advanced Circos” tool was employed to visualize the synteny map of the *NnCSL* family genes within each species, and the “Dual Synteny Plot for MCscanX” tool was used to visualize the interspecies synteny map.

### 4.9. RNA-Seq and Analysis

The Lotus petioles from LPA and SPA varieties, along with normal stamens (S), petalized stamens (SP), normal carpels (C), and petalized carpels (CP) of the lotus variety ‘Shen Nvzi’, were subjected to RNA extraction with three biological replicates. mRNA library construction and RNA-seq were performed using the DNBSEQ platform (BGI Co., Ltd., Shenzhen, China). To identify genes corresponding to the reads for each sample library, and the reads were aligned to the *N. nucifera* reference genome (GCF_000365185.1_Chinese_Lotus_1.1) using TopHat version 2.1.1. Gene expression levels were quantified as fragments per kilobase of exon per million fragments mapped (FPKM). Heatmaps for DEGs were generated using DEGseq version 1.60.0, applying criteria of *q*-value < 0.005 and |log^2^ (FPKM-LPA/FPKM-SPA)| > 1 or log^2^ (FPKM-SP/FPKM-S)| > 1 or log^2^ (FPKM-CP/FPKM-C)| > 1. For gene ontology (GO) term annotations, all *N. nucifera* genes were queried against the National Center for Biotechnology Information (NCBI) non-redundant (Nr) protein database using GOSeq version 1.58.0, with a corrected *p*-value threshold of <0.05. Finally, differentially expressed *NnCSL* genes were identified.

### 4.10. Nucleic Acid Isolation and qRT-PCR Analysis

Total RNA was isolated using the Eastep™ Super Total RNA Extraction Kit (Promega, Madison, WI, USA). First-strand cDNA synthesis was performed with 1 μg of total RNA in 20 μL reactions, using HiScript II Q RT SuperMix for qPCR (Vazyme, Nanjing, China) and oligo-dT (18)-MN primers following the manufacturer’s instructions. qRT-PCR was conducted with SYBR^®^ Green Realtime PCR Master Mix-Plus (Takara, Tokyo, Japan) under the following conditions: polymerase activation for 30 s at 95 °C, followed by 40 cycles of 15 s at 95 °C, 15 s at 60 °C, and 25 s at 72 °C. The *NnUBC* (*LOC104586755*) gene served as an internal control, and gene expression was normalized to this reference gene. All primers used in these assays are listed in Table S6, and each assay was carried out with three biological replicates.

### 4.11. Heatmap of Enriched Correlations Between Candidate NnCSL Genes and TFs

TFs data were extracted from the transcriptome information described in Section 4.9. Correlation analysis between candidate *NnCSL* genes and TFs was performed using the enrichment tools available on the Metware Cloud platform (https://cloud.metware.cn, accessed on 2 September 2024). This analysis generated a correlation enrichment heatmap, which was subsequently visually enhanced using the HeatMap tool in TBtools. Pearson correlation coefficients were calculated to assess the relationships between structural genes and transcription factors, employing the filtering criteria of correlation coefficients >0.9 or <−0.9 and *p* < 0.05.

## 5. Conclusions

**Figure 9:**
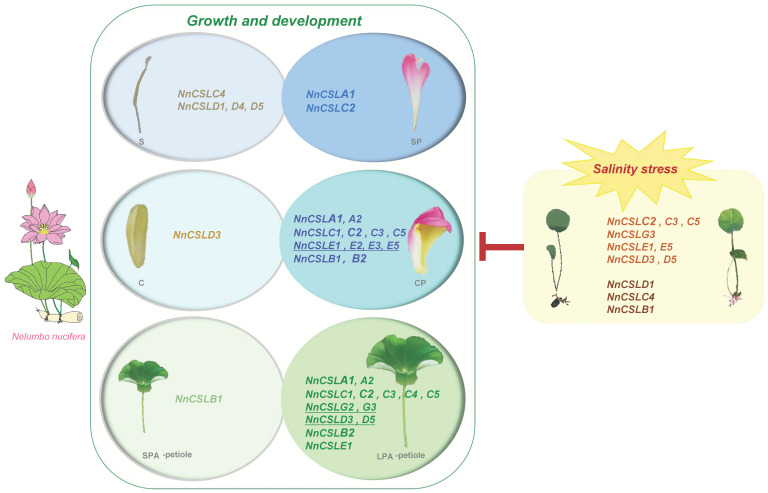
Hypothetical model of candidate *NnCSL* genes related to growth and development (petiole elongation, stamen petalization, and carpel petalization) and response to salinity stress. The model also highlights which *NnCSL* genes exhibit contrasting expression patterns in response to salinity stress compared to their roles in growth and development.

This study provides a comprehensive analysis of the 22 *NnCSL* genes identified within the whole genome of *N. nucifera*, which were categorized into six subfamilies: NnCSLA, NnCSLB, NnCSLC, NnCSLD, NnCSLE, and NnCSLG. Syntenic analyses revealed the genetic homologies between *N. nucifera* and other model plants such as *A. thaliana* and *O. sativa*, revealing not only the unique dicotyledonous characteristics of lotus but also its retention of certain monocotyledonous attributes. These findings underscore the profound implications for understanding gene conservation and variation across the plant kingdom. Beyond elucidating the structural and evolutionary relationships of these genes, the study also conducted an in-depth analysis of the promoter regions, highlighting the potential pivotal roles of *NnCSL* genes in plant growth and development, stress responses, and hormonal signaling pathways. Finally, we elucidated candidate *NnCSL* genes implicated in growth and development, specifically petiole elongation and floral petalization, as well as their responses to salinity stress in *N. nucifera* (Figure 9). These insights not only provide scientific evidence for the improvement of ornamental traits in lotus but also lay a foundation for the development of stress-tolerant breeding strategies. In summary, this research not only deepens our understanding of *NnCSL* genes in ancient dicotyledonous plants but also offers new perspectives and genetic resources for future investigations into plant gene functions and the development of novel breeding technologies.

## Figures and Tables

**Table 1:** The information of the *CSL* family genes in *N. nucifera*.

Gene Name	Gene ID	LOC	ORF (bp)	AA(aa)	Mw (KDa)	PI	Instability Index	Aliphatic Index	GRAVY	TMHs	Subcellular Localization
*NnCSLA1*	Nn2g13982.31	104612509	1638	546	61.906	8.84	43.55	101.39	0.208	5	Plasma membrane
*NnCSLA2*	Nn6g35072.4	104587567	1602	533	61.23	9.11	36.13	99.77	0.155	5	Plasma membrane
*NnCSLB1*	Nn1g03452.7	104588296	2304	767	85.923	6.47	45.6	96.62	0.067	8	Plasma membrane
*NnCSLB2*	Nn1g07221.3	104594052	2100	699	78.988	5.86	45.24	97.87	0.028	2	Plasma membrane
*NnCSLC1*	Nn1g08101.3	104589898	2079	692	79.686	8.63	43.58	101.01	0.022	5	Plasma membrane
*NnCSLC2*	Nn2g12599.4	104612365	2085	694	79.761	8.71	38.34	102.85	0.096	6	Plasma membrane
*NnCSLC3*	Nn3g16967.1	104595557	1989	662	75.846	9.13	37.11	106.19	0.194	5	Plasma membrane
*NnCSLC4*	Nn5g30690.8	104609129	2001	666	76.162	8.29	39.4	95.44	0.065	6	Plasma membrane
*NnCSLC5*	Nn8g40281.1	104605331	2100	699	80.629	8.81	36.45	100.3	0.072	6	Plasma membrane
*NnCSLD1*	Nn7g37641.1	104598378	3249	1082	120.315	6.87	43.63	80.6	−0.236	6	Plasma membrane
*NnCSLD2*	Nn1g00223.6	104604803	3444	1147	129.067	6.84	45.11	80.94	−0.211	8	Plasma membrane
*NnCSLD3*	Nn3g19380.2	104611451	3456	1151	129.122	6.64	42.26	82.17	−0.189	6	Plasma membrane
*NnCSLD4*	Nn2g12702.1	104599135	3378	1125	126.199	6.6	42.73	79.68	−0.215	8	Plasma membrane
*NnCSLD5*	Nn2g12438.4	104603242	2769	938	104.642	8.24	37.64	83.73	−0.16	8	Plasma membrane
*NnCSLE1*	Nn1g07535.2	104594270	2028	675	77.407	7.47	39.83	85.93	−0.02	8	Plasma membrane
*NnCSLE2*	Nn1g07538.2	104594272	2307	768	87.176	8.43	45.77	92.86	0.072	6	Plasma membrane
*NnCSLE3*	Nn5g27070.3	104594007	1425	474	53.794	8.92	43.05	96.88	0.07	4	Plasma membrane
*NnCSLE4*	Nn5g27071.10	109114471	2199	732	83.92	8.59	40.44	91.24	−0.044	6	Plasma membrane
*NnCSLE5*	Nn7g37928.3	104609477	2364	787	89.331	8.67	40.41	86.61	−0.068	8	Plasma membrane
*NnCSLG1*	Nn1g00277.23	104604823	2259	752	85.034	8.7	45.83	87.65	0.075	7	Plasma membrane
*NnCSLG2*	Nn1g02670.1	104610102	2034	677	76.588	8.73	51.21	102.54	0.189	8	Plasma membrane
*NnCSLG3*	Nn3g19586.4	104600820	2130	709	80.828	7.53	40.82	95.56	0.136	8	Plasma membrane

## Data Availability

The datasets supporting the results presented in this manuscript are included within the article (and its Supplementary Materials).

## Supplementary Materials

The following supporting information can be downloaded at: https://www.mdpi.com/article/10.3390/ijms252312531/s1.
