# Supplementary figures and images for: Genome-Wide Analysis of CSL Family Genes Involved in Petiole Elongation, Floral Petalization, and Response to Salinity Stress in Nelumbo nucifera
Source: Int J Mol Sci. 2024 Nov 22;25(23):12531. doi: 10.3390/ijms252312531 (PMC11641645; doi:10.3390/ijms252312531)

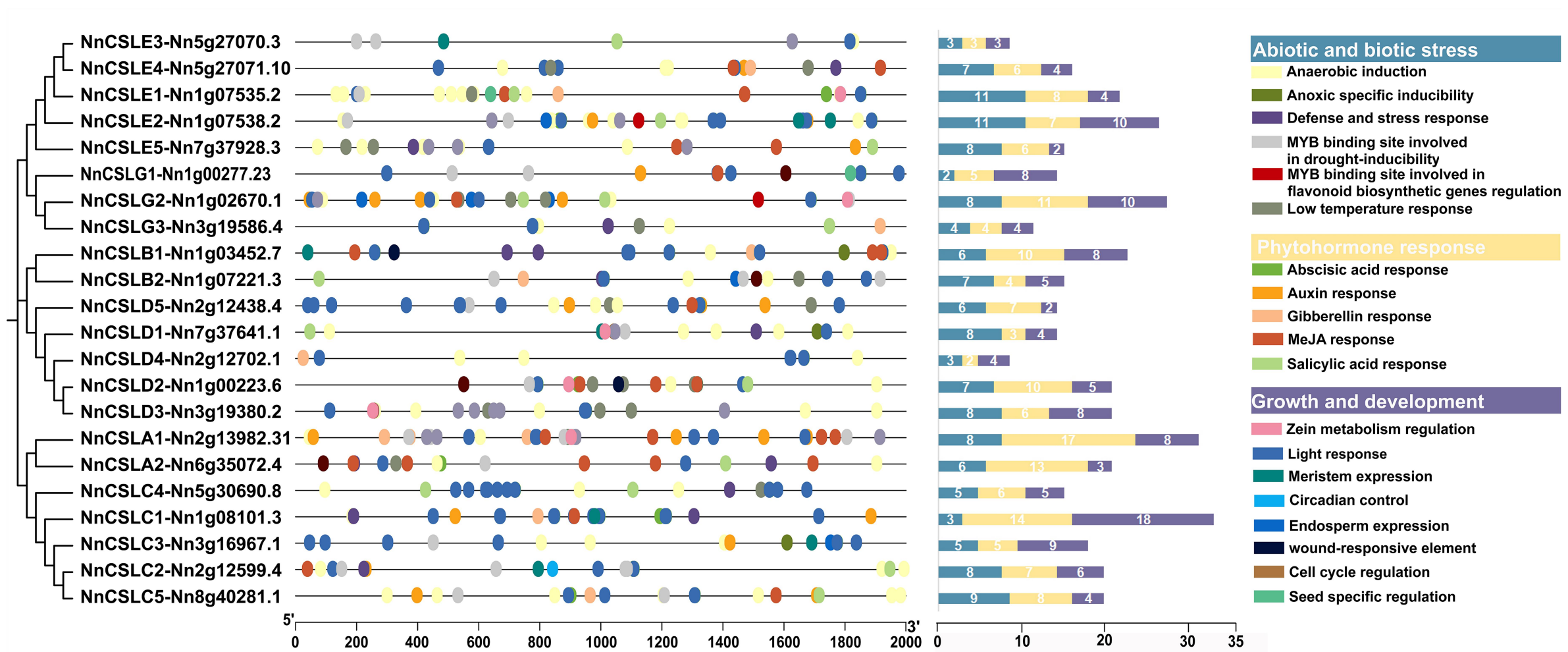

**Figure S1**

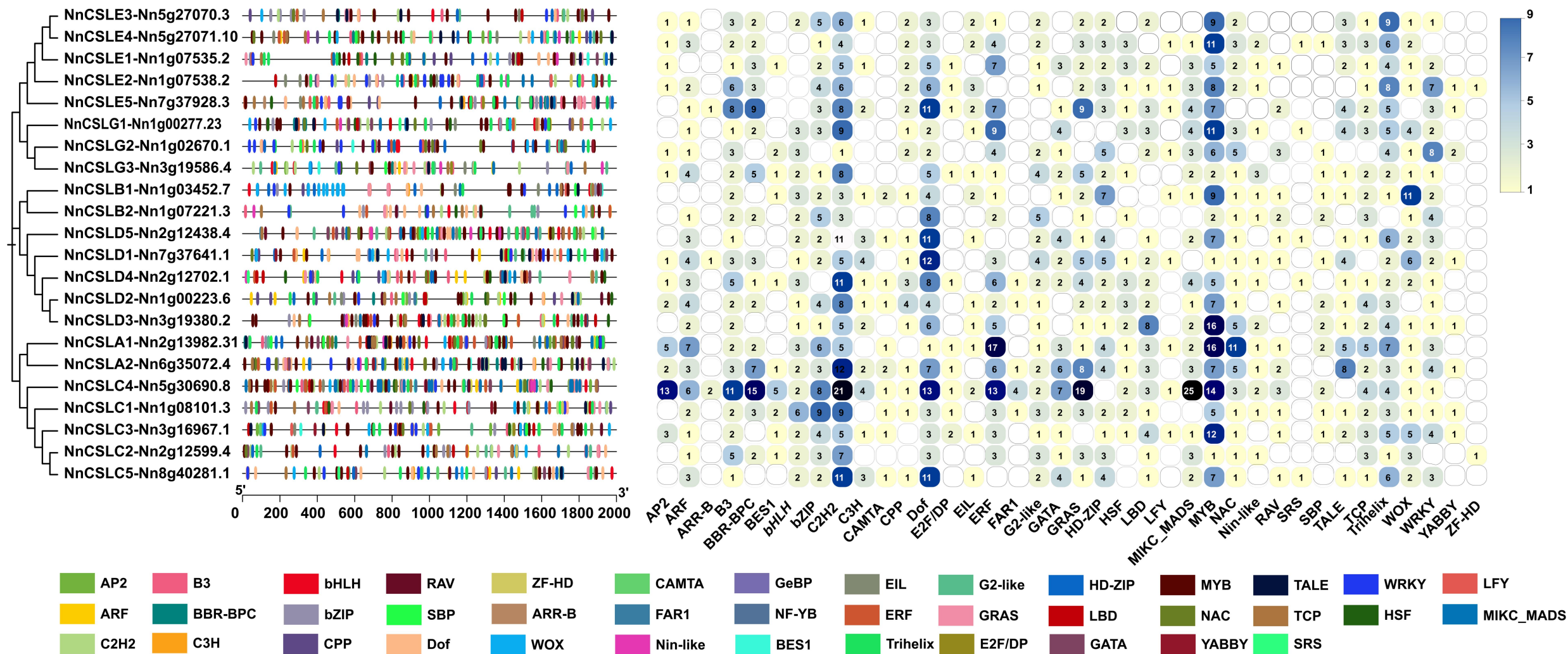

**Figure S2**

Supplement: Supplementary file 1 [file ijms-25-12531-s001.zip › ijms-3315947-supplementary figures.pdf]
